# Supplementary figures and images for: Clinical and economic burden of acute otitis media caused by Streptococcus pneumoniae in European children, after widespread use of PCVs–A systematic literature review of published evidence
Source: PLoS One. 2024 Apr 2;19(4):e0297098. doi: 10.1371/journal.pone.0297098 (PMC10986968; doi:10.1371/journal.pone.0297098)

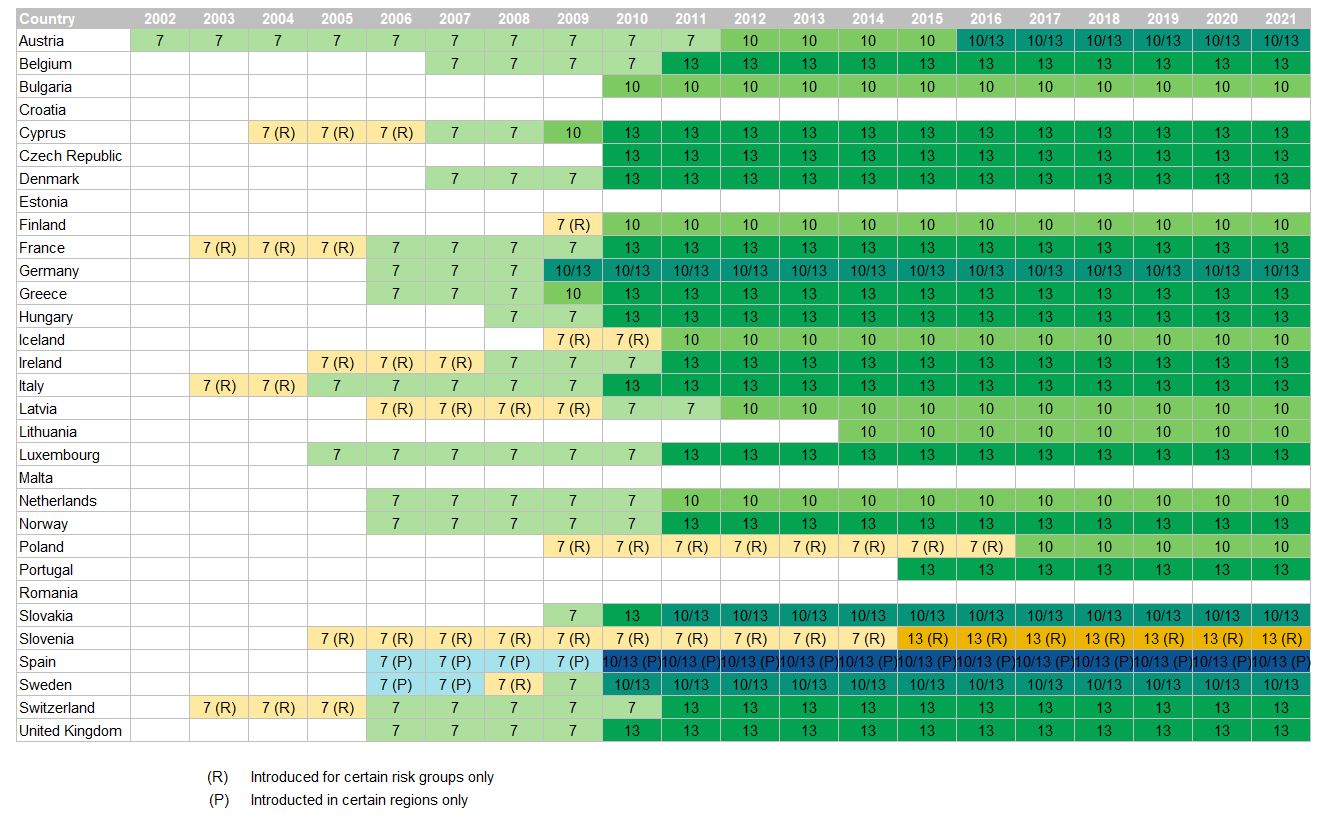

Supplement: S1 Fig — Notes: ®: Vaccine is not administered to the entire population, only to specific risk groups. (P): Vaccine is administered only in certain regions of the country. Sources: [9, 19–28]. (TIF) [file pone.0297098.s001.tif]
