# Supplementary material for: Clinical and economic burden of acute otitis media caused by Streptococcus pneumoniae in European children, after widespread use of PCVs–A systematic literature review of published evidence
Source: PLoS One. 2024 Apr 2;19(4):e0297098. doi: 10.1371/journal.pone.0297098 (PMC10986968; doi:10.1371/journal.pone.0297098)
Supplement: S1 Table — (DOCX) [file pone.0297098.s002.docx]

# Supporting information – Table S1

**S1 Table PubMed® search terms**

| **PubMed** | | |
| --- | --- | --- |
| **Objective** | **Search term (including filters for papers in English, published on or after 1 January 2011)** | **Preliminary number of search hits** |
| 1. Population: (Children up to 5 years of age, in Europe) | (child preschool OR infant OR pediatric OR (child* AND "5 years")) AND (Austria OR Belgium OR Bulgaria OR Croatia OR Cyprus OR Czech* OR Denmark OR England OR Estonia OR Europe* OR EU OR Finland OR France OR Germany OR Greece OR Holland OR Hungary OR Iceland OR Ireland OR Italy OR Latvia OR Lithuania OR Luxembourg OR Malta OR Netherlands OR Norway OR Poland OR Portugal OR Romania OR Scotland OR Slovakia OR Slovenia OR Spain OR Sweden OR Switzerland OR "United Kingdom" OR UK OR Wales) | 314,524 |
| 1. Exposure: AOM | "acute otitis media"[All Fields] OR "otitis media"[MeSH Terms] | 4,732 |
| 1. Epidemiology and etiology | ((inciden*[Title/Abstract] OR prevalen*[Title/Abstract]) AND (epidemiology)) OR "aetiologie"[TIAB] OR "aetiologies"[TIAB] OR "aetiology"[TIAB] OR "etiologies"[TIAB] OR "etiology"[TIAB] OR "causality"[TIAB] | 508,276 |
| 1. Burden (costs, resource use and HRQoL) | "cost of illness"[All Fields] OR "costs and cost analysis"[MeSH Terms] OR "cost analysis"[All Fields] OR "economic burden"[All Fields] OR "burden of disease"[All Fields] OR "health resources"[MeSH Terms] OR "health resources"[All Fields] OR "resource use"[All Fields] OR "burden"[All Fields] OR "Healthcare resource utilization"[All Fields] OR "expenditure"[All Fields] OR "indirect cost"[All Fields] OR "healthcare cost"[All Fields] OR "hospital cost"[All Fields] OR "hospital costs"[All Fields] OR "in-patient"[All Fields] OR "inpatient"[All Fields] OR "out-patient"[All Fields] OR "outpatient"[All Fields] OR "quality of life"[MeSH Terms] OR "quality of life"[All Fields] OR (("health-related"[All Fields] OR "health related"[All Fields]) AND "quality"[All Fields] AND "life"[All Fields]) OR "health-related quality of life"[All Fields] OR "health related quality of life"[All Fields] OR "hrqol"[All Fields] OR "Patient-reported outcomes"[All Fields] OR "EQ-5D"[All Fields] OR "SF-36"[All Fields] | 666,670 |
| 1. *S. pneumoniae*: serotype distribution and antibiotic resistance | (Streptococcus pneumoniae OR "S pneumoniae" OR pneumococc*) AND (serotype distribution OR antibiotic resistance OR Antimicrobial resistance OR Antimicrobial susceptibility OR antibiotic susceptibility) | 3,743 |
| 1. VCR for PCV | ((("vaccination coverage"[MeSH Terms] OR ("vaccination"[All Fields] AND "coverage"[All Fields]) OR "vaccination coverage"[All Fields]) AND "rate"[All Fields]) OR (("vaccin"[Supplementary Concept] OR "vaccin"[All Fields] OR "vaccination"[MeSH Terms] OR "vaccination"[All Fields] OR "vaccinable"[All Fields] OR "vaccinal"[All Fields] OR "vaccinate"[All Fields] OR "vaccinated"[All Fields] OR "vaccinates"[All Fields] OR "vaccinating"[All Fields] OR "vaccinations"[All Fields] OR "vaccination s"[All Fields] OR "vaccinator"[All Fields] OR "vaccinators"[All Fields] OR "vaccine s"[All Fields] OR "vaccined"[All Fields] OR "vaccines"[MeSH Terms] OR "vaccines"[All Fields] OR "vaccine"[All Fields] OR "vaccins"[All Fields]) AND "rate"[All Fields]) OR ("vaccin*"[All Fields] AND ("rate"[All Fields] OR ("coverage"[All Fields] OR "coverages"[All Fields]) OR ("frequency"[All Fields] OR "frequence"[All Fields] OR "frequences"[All Fields] OR "frequencies"[All Fields])))) AND ((("pneumococcal vaccines"[MeSH Terms] OR ("pneumococcal"[All Fields] AND "vaccines"[All Fields]) OR "pneumococcal vaccines"[All Fields] OR "pneumococcal"[All Fields]) AND ("vaccines, conjugate"[MeSH Terms] OR ("vaccines"[All Fields] AND "conjugate"[All Fields]) OR "conjugate vaccines"[All Fields] OR ("conjugate"[All Fields] AND "vaccine"[All Fields]) OR "conjugate vaccine"[All Fields])) OR "PCV"[All Fields]) | 1,403 |
| 1. Filters | Publication date since Jan 1st, 2011  Language: English | N/A |
| 1 AND 2 AND 3 – Epidemiology & Etiology | | 231 |
| 1 AND 2 AND 4 – Burden | | 196 |
| 1 AND 2 AND 5 – Serotypes | | 71 |
| 1 AND 2 AND 6 – VCRs | | 60 |
| ***Total hits*** | | ***558*** |
| **Embase** | |  |
| **No.** | **Query** | **Results** |
| #1 | 'child preschool'/exp OR 'child preschool' OR ('child' AND 'preschool') OR 'infant'/exp OR 'infant*' OR 'paediatric*' OR 'pediatrics'/exp OR ('child*' AND '5 years') | 2260473 |
| #2 | 'austria' OR 'belgium' OR 'bulgaria' OR 'croatia' OR 'cyprus' OR 'denmark' OR 'england' OR 'estonia' OR 'finland' OR 'france' OR 'germany' OR 'greece' OR 'holland' OR 'hungary' OR 'iceland' OR 'ireland' OR 'italy' OR 'latvia' OR 'lithuania' OR 'luxembourg' OR 'malta' OR 'netherlands' OR 'norway' OR 'poland' OR 'portugal' OR 'romania' OR 'scotland' OR 'slovakia' OR 'slovenia' OR 'spain' OR 'sweden' OR 'switzerland' OR 'united kingdom' OR 'uk' OR 'wales' OR 'austria'/exp OR 'belgium'/exp OR 'bulgaria'/exp OR 'croatia'/exp OR 'cyprus'/exp OR 'czech*' OR 'denmark'/exp OR 'england'/exp OR 'estonia'/exp OR 'europe*' OR 'eu' OR 'finland'/exp OR 'france'/exp OR 'germany'/exp OR 'greece'/exp OR 'holland'/exp OR 'hungary'/exp OR 'iceland'/exp OR 'ireland'/exp OR 'italy'/exp OR 'latvia'/exp OR 'lithuania'/exp OR 'luxembourg'/exp OR 'malta'/exp OR 'netherlands'/exp OR 'norway'/exp OR 'poland'/exp OR 'portugal'/exp OR 'romania'/exp OR 'scotland'/exp OR 'slovakia'/exp OR 'slovenia'/exp OR 'spain'/exp OR 'sweden'/exp OR 'switzerland'/exp OR 'united kingdom'/exp OR 'uk'/exp OR 'wales'/exp | 23028909 |
| #3 | 'acute otitis media' OR 'acute otitis media'/exp | 8078 |
| #4 | #1 AND #2 AND #3 | 2366 |
| #5 | ('inciden*':ti,ab OR 'prevalen*':ti,ab) AND 'epidemiology' OR 'aetiologie':ti,ab OR 'aetiologies':ti,ab OR 'aetiology':ti,ab OR 'etiologies':ti,ab OR 'etiology':ti,ab OR 'causality':ti,ab | 989578 |
| #6 | 'cost of illness' OR 'cost of illness'/exp OR 'costs' OR 'costs and cost analysis'/exp OR 'cost analysis' OR 'economic burden' OR 'economic burden'/exp OR 'burden of disease' OR 'burden of disease'/exp OR 'health resources'/exp OR 'health resources' OR 'resource use' OR 'burden' OR 'healthcare resource utilization' OR 'expenditure' OR 'indirect cost' OR 'healthcare cost' OR 'hospital cost*' OR 'inpatient' OR 'outpatient' OR 'quality of life'/exp OR 'quality of life' OR 'hrqol' OR 'patient-reported outcomes' OR 'eq-5d' OR 'sf-36' | 2114477 |
| #7 | ('streptococcus pneumoniae'/exp OR 'streptococcus pneumoniae') AND ('serotype prevalence'/exp OR 'serotype prevalence' OR 'antibiotic resistance'/exp OR 'antibiotic resistance' OR 'antibiotic sensitivity'/exp OR 'antibiotic sensitivity') | 14740 |
| #8 | (('vaccination coverage'/exp OR ('vaccination' AND 'coverage') OR 'vaccination coverage') AND 'rate' OR (('vaccin*' OR 'vaccin'/exp) AND ('rate' OR 'coverage' OR 'coverages' OR 'frequenc*'))) AND (('pneumococcal vaccines'/exp OR ('pneumococcal' AND 'vaccin*') OR 'pneumococcal vaccines' OR 'pneumococcal') AND ('vaccines, conjugate'/exp OR ('vaccines' AND 'conjugate') OR 'conjugate vaccines' OR ('conjugate' AND 'vaccine') OR 'conjugate vaccine') OR 'pcv') | 2788 |
| #9 | #4 AND #5 AND [english]/lim AND [2011-2021]/py | 161 |
| #10 | #4 AND #6 AND [english]/lim AND [2011-2021]/py | 301 |
| #11 | #4 AND #7 AND [english]/lim AND [2011-2021]/py | 87 |
| #12 | #4 AND #8 AND [english]/lim AND [2011-2021]/py | 103 |
| ***Total hits (#9 + #10 + #11 + #12)*** | | ***652*** |
| **Google searches** | | |
| *<Country name>* AND children AND (acute otitis media OR streptococcus pneumoniae) AND (economic burden OR cost analysis OR resource use OR hospitalization OR vaccine coverage rate OR epidemiology OR incidence OR prevalence OR serotype distribution OR antibiotic resistance OR antimicrobial resistance OR etiology) | | |
| ***Total hits (all countries combined)*** | | ***273*** |
| **Conference websites** | | |
| **Conference** | **Search term** | **Total hits** |
| ECCMID 2017-2021 | Acute otitis media | 6 |
|  | Streptococcus pneumoniae | 157 |
| ECCMID 2020 | Acute otitis media | 9 |
|  | Streptococcus pneumoniae | 144 |
| ESPID 2021 | Acute otitis media | 11 |
|  | Streptococcus pneumoniae | 38 |
| ESPID 2020 | Acute otitis media | 26 |
|  | Streptococcus pneumoniae | 48 |
| ESPID 2019 | Acute otitis media | 32 |
|  | Streptococcus pneumoniae | 48 |
| ESPID 2018 | Acute otitis media | 25 |
|  | Streptococcus pneumoniae | 61 |
| ESPID 2017 | Acute otitis media | 24 |
|  | Streptococcus pneumoniae | 57 |
| EAP 2017 | Acute otitis media | 3 |
|  | Streptococcus pneumoniae | 1 |
| EAP 2019 | Acute otitis media | 6 |
|  | Streptococcus pneumoniae | 5 |
| EAP 2020 | Acute otitis media | 1 |
|  | Streptococcus pneumoniae | 6 |
| ***Total hits (all conferences)*** | | ***708*** |
