# Supplementary material for: Clinical and economic burden of acute otitis media caused by Streptococcus pneumoniae in European children, after widespread use of PCVs–A systematic literature review of published evidence
Source: PLoS One. 2024 Apr 2;19(4):e0297098. doi: 10.1371/journal.pone.0297098 (PMC10986968; doi:10.1371/journal.pone.0297098)
Supplement: S2 Table — (DOCX) [file pone.0297098.s003.docx]

# Supporting information – Table S2

**S2 Table Type of data extracted from included records**

| **Feature** | **Variable (format)** |
| --- | --- |
| General information | - Title (text) - Authors (text) - Year (YYYY) - Country(ies) (text) - Population (age groups, description in text) - Sample size (n) |
| Epidemiological data | - Incidence of AOM (time interval, measurement, estimate, confidence intervals (CIs))   - Hospitalization rate associated to AOM - Prevalence of AOM (time interval, measurement, estimate, CIs) - Etiology (causes, estimates of shares, CIs) |
| Serotype and antibiotic data for *S. pneumoniae* | - Serotype distribution (bacteria type(s), serotype, estimates, CIs) - Patterns of antibiotic resistance and susceptibility (bacteria, resistance or susceptibility, type of antibiotic, measurement, risk rate, CIs) |
| Economic data | - Resource use (event type, currency, types of resources, estimates, CIs) - Costs (event type, direct/indirect, types of costs, estimates, CIs) - Quality of life   - Utility value scores (event description [e.g. hearing loss, tube, etc.], values, CIs, instrument if available) |
