# Supplementary material for: Clinical and economic burden of acute otitis media caused by Streptococcus pneumoniae in European children, after widespread use of PCVs–A systematic literature review of published evidence
Source: PLoS One. 2024 Apr 2;19(4):e0297098. doi: 10.1371/journal.pone.0297098 (PMC10986968; doi:10.1371/journal.pone.0297098)
Supplement: S3 Table — (DOCX) [file pone.0297098.s004.docx]

# Supporting information – Table S3

**S3 Table List of included records**

| **Identifier** | **Title** | **Year of data collection** | **Country** | **ROB tool** | **ROB rating** |
| --- | --- | --- | --- | --- | --- |
| Allemann et al 2017 [77] | Pneumococcal carriage and serotype variation before and after introduction of pneumococcal conjugate vaccines in patients with acute otitis media in Switzerland | 2004-2015 | Switzerland | ISPOR-AMCP-NPC | High quality |
| Alonso et al 2013 [65] | Dynamics of Streptococcus pneumoniae serotypes causing acute otitis media isolated from children with spontaneous middle-ear drainage over a 12-year period (1999-2010) in a region of northern Spain | 1999 – 2010 | Spain | ISPOR-AMCP-NPC | Medium quality |
| Angoulvant et al 2015 [15] | Trends in antibiotic resistance of Streptococcus pneumoniae and Haemophilus influenzae isolated from nasopharyngeal flora in children with acute otitis media in France before and after 13 valent pneumococcal conjugate vaccine introduction | Nov 2006 – Jun 2013 | France | ISPOR-AMCP-NPC | High quality |
| Ansaldi et al 2020 [79] | Estimating the Clinical and Economic Impact of Switching from the 13-Valent Pneumococcal Conjugate Vaccine (PCV13) to the 10-Valent Pneumococcal Conjugate Vaccine (PCV10) in Italy | Epidemiological: 2007 – 2010  Economic: 2013 | Italy | ECOBIAS | Medium quality |
| Barbieri et al 2020 [102] | A retrospective database analysis to estimate the burden of acute otitis media in children 0-14 years in the Veneto region, Italy | 01 Jan 2010 – 31 Dec 2017 | Italy | ISPOR-AMCP-NPC | High quality (Abstract) |
| Besednjak-Kocijancic et al 2017 [103] | Effect of probiotic microorganism lactobacillus reuteri on the occurrence of acute otitis media | Not available | Slovenia | ISPOR-AMCP-NPC | Medium quality (Abstract) |
| Blank et al 2012 [104] | Cost-effectiveness of 13-valent pneumococcal conjugate vaccine in Switzerland | Epidemiological: 2007 – 2008  Economic: 2004 | Switzerland | ECOBIAS | Medium quality |
| Boonacker et al 2011 [80] | Cost effectiveness of pneumococcal conjugate vaccination against acute otitis media in children: a review | Until 18 Feb 2010* | Spain, Sweden, Netherland, Germany, Switzerland, Finland, Norway | ECOBIAS | Medium quality |
| By et al 2012 [105] | Comparing health outcomes and costs of general vaccination with pneumococcal conjugate vaccines in Sweden: a Markov model | 2012 | Sweden | ECOBIAS | Medium quality |
| Caeymaex et al 2014 [66] | Characteristics and outcomes of acute otitis media in children carrying Streptococcus pneumoniae or Haemophilus influenzae in their nasopharynx as a single otopathogen after introduction of the heptavalent pneumococcal conjugate vaccine | October 2007 – May 2010 | France | ISPOR-AMCP-NPC | Medium quality |
| Castiglia et al 2017 [36] | Overall effectiveness of pneumococcal conjugate vaccines: An economic analysis of PHiD-CV and PCV-13 in the immunization of infants in Italy | 2017 | Italy | ECOBIAS | High quality |
| Chapman et al 2020 [26] | Ten-year public health impact of 13-valent pneumococcal conjugate vaccination in infants: A modelling analysis | 2000 – 2015 | Europe | ISPOR-AMCP-NPC | High quality |
| Chen et al 2019 [106] | Effect and cost-effectiveness of pneumococcal conjugate vaccination: a global modelling analysis | 2019 | Europe | ECOBIAS | Medium quality |
| Cohen et al 2011 [107] | Risk factors for serotype 19A carriage after introduction of 7-valent pneumococcal vaccination | Nov 2006 – Jun 2009 | France | ISPOR-AMCP-NPC | High quality |
| Cohen et al 2012 [67] | Nasopharyngeal flora in children with acute otitis media before and after implementation of 7 valent pneumococcal conjugate vaccine in France | 1993 – 2000; 2006 - 2009 | France | ISPOR-AMCP-NPC | High quality |
| Cohen et al 2015 [59] | A 13-year survey of pneumococcal nasopharyngeal carriage in children with acute otitis media following PCV7 and PCV13 implementation | Oct 2001 – Jun 2014 | France | ISPOR-AMCP-NPC | High quality |
| Cohen et al 2016 [50] | The multifaceted impact of pneumococcal conjugate vaccine implementation in children in France between 2001 to 2014 | 2001 – 2014 (Oct to Jun) | France | ROBIS | Low quality |
| Cohen et al 2020 A [76] | 18 years of surveillance of nasopharyngeal pneumococcal carriage before, during, and after PCV7 then PCV13 implementation in children with acute otitis media | 2001 – 2019 | France | ISPOR-AMCP-NPC | Medium quality (Abstract) |
| Cohen et al 2020 B [78] | Potential serotype coverage of third generation PCVs in Israel and France in children 6-23 months old | Not available | France | ISPOR-AMCP-NPC | High quality (Abstract) |
| Couloigner et al 2012 [68] | Pathogens implicated in acute otitis media failures after 7-valent pneumococcal conjugate vaccine implementation in France: distribution, serotypes, and resistance levels | May 2007 – Apr 2009 | France | ISPOR-AMCP-NPC | Medium quality |
| Damm et al 2015 [108] | Public health impact and cost-effectiveness of intranasal live attenuated influenza vaccination of children in Germany | 2015 | Germany | ECOBIAS | High quality |
| del Castillo-Aguas et al 2017 [109] | Infectious morbidity and resource use in children under 2 years old at childcare centres | 01 Apr 2009 – 31 Mar 2012 | Spain | ISPOR-AMCP-NPC | High quality |
| Delgleize et al 2016 [110] | Cost-effectiveness analysis of routine pneumococcal vaccination in the UK: a comparison of the PHiD-CV vaccine and the PCV-13 vaccine using a Markov model | 2016 | UK | ECOBIAS | High quality |
| Díez-Gandía et al 2018 [110] | Real world data shows inadequate use of antibiotics and lack of impact of pneumococcal conjugate vaccine in acute otitis media in Spain | Not available | Spain | ISPOR-AMCP-NPC | Low quality (Abstract) |
| Durando et al 2012 [111] | Improving the protection against Streptococcus pneumoniae with the new generation 13-valent pneumococcal conjugate vaccine | Not available | Italy | NA | NA |
| Edmondson-Jones et al 2021 A [39] | Impact of pneumococcal conjugate vaccines on healthcare utilization and direct costs for otitis media in children ?2 years of age in two Swedish regions | 2005 – 2013 | Sweden | ISPOR-AMCP-NPC | High quality |
| Edmondson-Jones et al 2021 B [112] | The effect of pneumococcal conjugate vaccines on otitis media from 2005 to 2013 in children aged ?5 years: a retrospective cohort study in two Swedish regions | 01 Jan 1999 – 31 Dec 2013 | Sweden | ISPOR-AMCP-NPC | High quality |
| Ekinci et al 2021 [73] | Streptococcus pneumoniae Serotypes Carried by Young Children and Their Association With Acute Otitis Media During the Period 2016–2019 | 2016 – 2018 (Nov to May) | Belgium | ISPOR-AMCP-NPC | Medium quality |
| Esposito et al 2016 [113] | Impact of Streptococcus pneumoniae in acute otitis media (AOM) with spontaneous tympanic membrane perforation (STMP): Serotype distribution 4 years after 13-valent pneumococcal conjugate vaccine (PCV13) introduction | Not available | Italy | ISPOR-AMCP-NPC | High quality (Abstract) |
| Eythorsson et al 2018 [114] | Decreased Acute Otitis Media With Treatment Failure After Introduction of the Ten-valent Pneumococcal Haemophilus influenzae Protein D Conjugate Vaccine | 2008 - 2015 | Iceland | ISPOR-AMCP-NPC | High quality |
| Falup-Pecurariu et al 2013 [55] | Pneumococcal acute otitis media in infants and children in central Romania, 2009-2011: microbiological characteristics and potential coverage by pneumococcal conjugate vaccines | 2009 - 2011 | Romania | ISPOR-AMCP-NPC | Medium quality |
| Fenoll et al 2011 [115] | Increase in serotype 19A prevalence and amoxicillin non-susceptibility among paediatric Streptococcus pneumoniae isolates from middle ear fluid in a passive laboratory-based surveillance in Spain, 1997-2009 | Jan 1997 – Jun 2009 | Spain | ISPOR-AMCP-NPC | Low quality |
| Fortanier et al 2015 [37] | Parent-Reported symptoms of acute otitis media during the first year of Life: What is beneath the surface? | From 2008 | Netherlands | ISPOR-AMCP-NPC | Medium quality |
| Fortanier et al 2019 [116] | Does pneumococcal conjugate vaccination affect onset and risk of first acute otitis media and recurrences? A primary care-based cohort study | 2004 - 2015 | Netherlands | ISPOR-AMCP-NPC | High quality |
| Fortunato et al 2015 [43] | Impact of Pneumococcal Conjugate Universal Routine Vaccination on Pneumococcal Disease in Italian Children | 2001 - 2011 | Italy | ISPOR-AMCP-NPC | Medium quality |
| Gisselsson-Solen et al 2017 [41] | Trends in Otitis Media Incidence After Conjugate Pneumococcal Vaccination: A National Observational Study | 2005 – 2014 | Sweden | ISPOR-AMCP-NPC | Medium quality |
| Górska-Kot et al 2019 [53] | Characterization of acute otitis media otopathogens before the introduction of the pneumococcal conjugated vaccine into the national immunization program in Poland | 2010 – 2016 | Poland | ISPOR-AMCP-NPC | Medium quality |
| Gouveia et al 2017 [117] | Cost-effectiveness of the 13-valent Pneumococcal Conjugate Vaccine in Children in Portugal | 2017 | Portugal | ECOBIAS | High quality |
| Grall et al 2011 [118] | Epidemiology of Streptococcus pneumoniae in France before introduction of the PCV-13 vaccine | Jan 2009 – Dec 2009 | France | ISPOR-AMCP-NPC | Medium quality |
| Grevers et al 2012 [69] | Identification and characterization of the bacterial etiology of clinically problematic acute otitis media after tympanocentesis or spontaneous otorrhea in German children | Nov 2008 – Apr 2010 | Germany | ISPOR-AMCP-NPC | Medium quality |
| Heidemann et al 2013 [88] | The Otitis Media-6 questionnaire: psychometric properties with emphasis on factor structure and interpretability | 15 Feb 2011 – 28 Feb 2012 | Denmark | ISPOR-AMCP-NPC | High quality |
| Heidemann et al 2014 [91] | Caregiver Quality of Life and Daily Functioning in Relation to Ventilating Tube Treatment | Feb-2011 – Mar 2012 | Denmark | ISPOR-AMCP-NPC | Medium quality |
| Heidemann et al 2015 [90] | Quality-of-Life Differences among Diagnostic Subgroups of Children Receiving Ventilating Tubes for Otitis Media | Feb-2011 – Mar 2012 | Denmark | ISPOR-AMCP-NPC | High quality |
| Holl et al 2015 [93] | The Impact of Childhood Acute Otitis Media on Parental Quality of Life in a Prospective Observational Cohort Study | Jul 2008 – Jan 2009 | Italy, Germany, UK, Spain, Sweden | ISPOR-AMCP-NPC | Medium quality |
| Hu et al 2020 [31] | Clinical and economic burden of recurrent AOM in children in Germany from 2012-2017 | 2012 – 2017 | Germany | ISPOR-AMCP-NPC | High quality (abstract) |
| Imöhl et al 2021 [44] | Bacterial Spectrum of Spontaneously Ruptured Otitis Media in a 7-Year, Longitudinal, Multicenter, Epidemiological Cross-Sectional Study in Germany | Oct 2008 – Oct 2015 | Germany | ISPOR-AMCP-NPC | High quality |
| Indius et al 2018 [119] | Middle ear disease in Danish toddlers attending nursery day-care - Applicability of OM-6, disease specific quality of life and predictors for middle ear symptoms | 24 Feb 2014 – 28 Mar 2014 | Denmark | ISPOR-AMCP-NPC | High quality |
| Jinhage et al 2021 | Nasopharyngeal cultures in children with AOM – A retrospective study on bacteriological findings and impact on management | 01 Jan 2018 – 31 Dec 2018 | Sweden | ISPOR-AMCP-NPC | Medium quality |
| Kempf et al 2015 | Decline in antibiotic resistance and changes in the serotype distribution of Streptococcus pneumoniae isolates from children with acute otitis media; a 2001-2011 survey by the French Pneumococcal Network | 2001 – 2011 | France | ISPOR-AMCP-NPC | High quality |
| Klok et al 2013 [120] | Cost-effectiveness of a 10- versus 13-valent pneumococcal conjugate vaccine in Denmark and Sweden | 2013 | Denmark, Sweden | ECOBIAS | High quality |
| Knerer et al 2012 [99] | Health and economic impact of PHiD-CV in Canada and the UK: a Markov modelling exercise | 2012 | UK | ECOBIAS | High quality |
| Koliou et al 2018 [121] | Risk factors for carriage of Streptococcus pneumoniae in children | 2007 – 2008 | Cyprus | NA | NA |
| Korona-Glowniak et al 2018 [54] | Resistant Streptococcus pneumoniae strains in children with acute otitis media– high risk of persistent colonization after treatment | 2010 – 2014 | Poland | ISPOR-AMCP-NPC | Medium quality |
| Kostenniemi et al 2018 | Reductions in otitis and other respiratory tract infections following childhood pneumococcal vaccination | 01 Jan 2005 – 31 Dec 2014 | Sweden | ISPOR-AMCP-NPC | High quality |
| Kuhlmann et al 2017 [122] | Modeling the cost-effectiveness of infant vaccination with pneumococcal conjugate vaccines in Germany | 2017 | Germany | ECOBIAS | High quality |
| Kvaerner et al 2013 [123] | Hospitalization for acute otitis media as a useful marker for disease severity | 1999 - 2006 | Norway | ISPOR-AMCP-NPC | Medium quality |
| Laulajainen-Hongisto et al 2014 [46] | Bacteriology in relation to clinical findings and treatment of acute mastoiditis in children | 2003 – 2012 | Finland | ISPOR-AMCP-NPC | Medium quality |
| Laulajainen-Hongisto et al 2015 [47] | Children hospitalized due to acute otitis media: how does this condition differ from acute mastoiditis? | 2003 – 2012 | Finland | ISPOR-AMCP-NPC | Low quality |
| Levy et al 2019 [51] | Bacterial causes of otitis media with spontaneous perforation of the tympanic membrane in the era of 13 valent pneumococcal conjugate vaccine | 2015 – 2018 | France | ISPOR-AMCP-NPC | Medium quality |
| Liese et al 2011 [82] | The clinical and economic burden of acute otitis media: A large prospective observational cohort study in Europe | Not available | Germany, Italy, Spain, Sweden and the UK | ISPOR-AMCP-NPC | Low quality (Abstract) |
| Liese et al 2014 [124] | Incidence and clinical presentation of acute otitis media in children aged <6 years in European medical practices | Jul 2008 – Jan 2009 | Germany, Italy, Spain, Sweden, UK | ISPOR-AMCP-NPC | High quality |
| Littorin et al 2016 [125] | Reduction of Streptococcus pneumoniae in upper respiratory tract cultures and a decreased incidence of related acute otitis media following introduction of childhood pneumococcal conjugate vaccines in a Swedish county | 2007 - 2013 | Sweden | ISPOR-AMCP-NPC | Medium quality |
| Lixandru et al 2017 [56] | Streptococcus pneumoniae Serotypes and Antibiotic Susceptibility Patterns in Middle Ear Fluid Isolates During Acute Otitis Media and Nasopharyngeal Isolates During Community-acquired Alveolar Pneumonia in Central Romania | 2009 - 2014 | Romania | ISPOR-AMCP-NPC | Medium quality |
| Macaj et al 2019 [75] | Streptococcus pneumoniae as a cause of acute otitis media in Slovak children in pneumococcal vaccination era | Jan 2016 – Jun 2017 | Slovakia | ISPOR-AMCP-NPC | Low quality (Abstract) |
| Magnus et al 2012 [126] | Decline in early childhood respiratory tract infections in the Norwegian mother and child cohort study after introduction of pneumococcal conjugate vaccination | Not available | Norway | ISPOR-AMCP-NPC | High quality |
| Marchisio et al 2012 [127] | Burden of acute otitis media in primary care pediatrics in Italy: a secondary data analysis from the Pedianet database | Jan 2003 – Dec 2007 | Italy | ISPOR-AMCP-NPC | Medium quality |
| Marchisio et al 2017 A [52] | Prospective evaluation of the aetiology of acute otitis media with spontaneous tympanic membrane perforation | =1 May 2015 – 30 Apr 2016 | Italy | ISPOR-AMCP-NPC | Medium quality |
| Marchisio et al 2017 B [74] | Serotypes not Included in 13-Valent Pneumococcal Vaccine as Causes of Acute Otitis Media with Spontaneous Tympanic Membrane Perforation in a Geographic Area with High Vaccination Coverage | 01 Apr 2015 – 31 Mar 2016 | Italy | ISPOR-AMCP-NPC | Medium quality |
| Martinelli et al 2014 [42] | Towards the 13-valent pneumococcal conjugate universal vaccination: Effectiveness in the transition era between PCV7 and PCV13 in Italy, 2010-2013 | May 2010 – Jan 2013 | Italy | ISPOR-AMCP-NPC | Medium quality |
| Monasta et al 2012 [4] | Burden of disease caused by otitis media: Systematic review and global estimates | Reviewed period: 1980 – 2008 | Europe | ROBIS | Medium quality |
| Morales et al 2018 [128] | Changes in the serotype distribution of Streptococcus pneumoniae causing otitis media after PCV13 introduction in Spain | 2008 – 2016, | Spain | ISPOR-AMCP-NPC | Medium quality |
| Ochoa-Gondar et al 2015 [129] | Epidemiology of Streptococcus pneumoniae causing acute otitis media among children in Southern Catalonia throughout 2007-2013: Incidence, serotype distribution and vaccine's effectiveness | 01 Jan 2007 – 31 Dec 2013 | Spain | ISPOR-AMCP-NPC | Medium quality |
| Ouldali et al 2018 [61] | Changes in bacterial nasopharyngeal carriage in children with acute otitis media following PCV13 implementation: a time series analysis of a 10-year multicenter prospective survey | Nov 2006 – Mar 2017 | France | ISPOR-AMCP-NPC | High quality (abstract) |
| Ouldali et al 2019 [62] | Pneumococcal susceptibility to antibiotics in carriage: a 17-year time series analysis of the adaptive evolution of non-vaccine emerging serotypes to a new selective pressure environment | 2001 – 2018 | France | ISPOR-AMCP-NPC | High quality |
| Pérez-Trallero et al 2012 [70] | Decline and rise of the antimicrobial susceptibility of Streptococcus pneumoniae isolated from middle ear fluid in children: influence of changes in circulating serotypes | 1999 – 2010 | Spain | ISPOR-AMCP-NPC | Medium quality |
| Prins-Van Ginkel et al 2017 [38] | Acute otitis media during infancy: Parent-reported incidence and modifiable risk factors | Oct 2007 – Dec 2012 | Netherlands | ISPOR-AMCP-NPC | High quality |
| Pugh et al 2020 [130] | Estimating the Impact of Switching from a Lower to Higher Valent Pneumococcal Conjugate Vaccine in Colombia, Finland, and The Netherlands: A Cost-Effectiveness Analysis | 2020 | Finland, Netherlands | ECOBIAS | Medium quality |
| Pumarola et al 2013 [71] | Microbiology of bacteria causing recurrent acute otitis media (AOM) and AOM treatment failure in young children in Spain: shifting pathogens in the post-pneumococcal conjugate vaccination era | May 2008 - Mar 2010 | Spain | ISPOR-AMCP-NPC | Medium quality |
| Pumarola et al 2016 [57] | Bacterial etiology of acute otitis media in Spain in the post-pneumococcal conjugate vaccine era | Feb 2009 – May 2012 | Spain | ISPOR-AMCP-NPC | High quality |
| Quirk et al 2018 [63] | Effect of Vaccination on Pneumococci Isolated from the Nasopharynx of Healthy Children and the Middle Ear of Children with Otitis Media in Iceland | 2009 - 2017 | Iceland | ISPOR-AMCP-NPC | High quality |
| Radzikowski et al 2011 [131] | Does nasopharyngeal bacterial flora predict etiology of acute otitis media in children? | Not available | Poland | ISPOR-AMCP-NPC | Low quality |
| Robberstad et al 2011 [132] | Economic evaluation of second-generation pneumococcal conjugate vaccines in Norway | 2011 | Norway | ECOBIAS | High quality |
| Rybak et al 2018 [60] | Antibiotic Resistance of Potential Otopathogens Isolated From Nasopharyngeal Flora of Children With Acute Otitis Media Before, During and After Pneumococcal Conjugate Vaccines Implementation | 2001 - 2016 | France | ISPOR-AMCP-NPC | High quality |
| Scholz et al 2019 [133] | Epidemiology and cost of seasonal influenza in Germany - a claims data analysis | 2012 – 2014 | Germany | ISPOR-AMCP-NPC | Medium quality |
| Setchanova et al 2013 [134] | Microbiological characterization of Streptococcus pneumoniae and non-typeable Haemophilus influenzae isolates as primary causes of acute otitis media in Bulgarian children before the introduction of conjugate vaccines | 1994 – 2011 | Bulgaria | ISPOR-AMCP-NPC | Medium quality |
| Setchanova et al 2017 [135] | Serotype changes and antimicrobial nonsusceptibility rates of invasive and non-invasive Streptococcus pneumoniae isolates after implementation of 10-valent pneumococcal nontypeable Haemophilus influenzae protein D conjugate vaccine (PHiD-CV) in Bulgaria | May 2011 – May 2016 | Bulgaria | ISPOR-AMCP-NPC | Medium quality |
| Shiri et al 2019 [19] | Pneumococcal Disease: A Systematic Review of Health Utilities, Resource Use, Costs, and Economic Evaluations of Interventions | Reviewed period: 01 Jan 1990 – 30 Nov 2016 | Sweden | ROBIS | Medium quality |
| Sigurdsson et al 2015 [27] | Decreased Incidence of Respiratory Infections in Children After Vaccination with Ten-valent Pneumococcal Vaccine | 01 Jan 2008 – 31 Dec 2013 | Iceland | ISPOR-AMCP-NPC | High quality |
| Sigurdsson et al 2018 [28] | Reduction in All-Cause Acute Otitis Media in Children <3 Years of Age in Primary Care Following Vaccination With 10-Valent Pneumococcal Haemophilus influenzae Protein-D Conjugate Vaccine: A Whole-Population Study | 01 Jan 2005 – 31 Dec 2015 | Iceland | ISPOR-AMCP-NPC | High quality |
| Sigurdsson et al 2020 [34] | Impact of the 10-valent pneumococcal conjugate vaccine on hospital admissions in children under three years of age in Iceland | 01 Jan 2005 – 31 Dec 2016 | Iceland | ISPOR-AMCP-NPC | High quality |
| Sillanpää et al 2016 A [49] | Antibiotic resistance in pathogens causing acute otitis media in Finnish children | Sep 2010 – Dec 2011 | Finland | ISPOR-AMCP-NPC | Medium quality |
| Sillanpää et al 2017 [48] | Next-Generation Sequencing Combined with Specific PCR Assays To Determine the Bacterial 16S rRNA Gene Profiles of Middle Ear Fluid Collected from Children with Acute Otitis Media | Sep 2010 – Dec 2011 | Finland | ISPOR-AMCP-NPC | Medium quality |
| Simões et al 2019 [45] | Multiple bacterial species are more often present in recurrent acute otitis media (rAOM) | Dec 2013 – Apr 2016, | Portugal | ISPOR-AMCP-NPC | High quality (abstract) |
| Speets et al 2011 [83] | Use of medical resources and indirect costs of otitis media in Sweden | Feb 2009 | Sweden | ISPOR-AMCP-NPC | Medium quality |
| Strutton et al 2012 [136] | Cost-effectiveness of 13-valent pneumococcal conjugate vaccine: Germany, Greece, and The Netherlands | 2012 | Germany, Greece, Netherlands | ECOBIAS | Medium quality |
| Sveinsdóttir et al 2019 [33] | The effect of the 10-valent pneumococcal nontypeable Haemophilus influenzae protein D conjugate vaccine on H. Influenzae in healthy carriers and middle ear infections in Iceland | 2009 and 2012 – 2017 | Iceland | ISPOR-AMCP-NPC | Medium quality |
| Toivonen et al 2016 B [137] | Rhinovirus Infections in the First 2 Years of Life | Jan 2008 – Apr 2010 | Finland | ISPOR-AMCP-NPC | High quality |
| Tóthpál et al 2012 [138] | Changes in the serotypes of Hungarian pneumococci isolated mainly from invasive infections: A review of all available data between 1988 and 2011 | Not available | Hungary | NA | NA |
| Tyrstrup et al 2017 [139] | Children with respiratory tract infections in Swedish primary care; prevalence of antibiotic resistance in common respiratory tract pathogens and relation to antibiotic consumption | 01 Nov 2013 30 Apr 2014 and 01 Nov 2014 – 30 Apr 2015 | Sweden | ISPOR-AMCP-NPC | Medium quality |
| Uijen et al 2011 [140] | ENT problems in Dutch children: trends in incidence rates, antibiotic prescribing and referrals 2002-2008 | 2002 – 2008 | Netherlands | ISPOR-AMCP-NPC | Medium quality |
| Uitti et al 2015 [141] | Role of Nasopharyngeal Bacteria and Respiratory Viruses in Acute Symptoms of Young Children | 2006 - 2008 | Finland | ISPOR-AMCP-NPC | Medium quality |
| Usonis et al 2016 [30] | Incidence of acute otitis media in children below 6 years of age seen in medical practices in five East European countries | Jun 2011 – Jan 2013 | Estonia, Lithuania, Poland, Romania, Slovenia | ISPOR-AMCP-NPC | Medium quality |
| van Brink et al 2019 [92] | Quality of life in Swedish children receiving grommets – An analysis of pre- and postoperative results based on a national quality register | 2010 – Oct 2016 | Sweden | ISPOR-AMCP-NPC | Medium quality |
| van Uum et al 2021 [81] | Cost of childhood acute otitis media in primary care in the Netherlands: economic analysis alongside a cluster randomized controlled trial | Feb 2015 – May 2018 | Netherlands | ECOBIAS | High quality |
| Vučina et al 2015[142] | Cost-effectiveness of pneumococcal conjugate vaccination in Croatia | 2015 | Croatia | ECOBIAS | Medium quality |
| Wouters et al 2018 [143] | Nasopharyngeal s. pneumoniae carriage and density in Belgian infants after 9 years of pneumococcal conjugate vaccine programme | 2016 – 2018 (Oct to Jun. In 2016, from Jan to Jun) | Belgium | ISPOR-AMCP-NPC | High quality |
| Wouters et al 2019 A [58] | Follow-up of serotype distribution and antimicrobial susceptibility of Streptococcus pneumoniae in child carriage after a PCV13-to-PCV10 vaccine switch in Belgium | Jan 2016 – Jun 2017 | Belgium | ISPOR-AMCP-NPC | High quality |
| *Date of literature search |  |  |  |  |  |
