# Supplementary material for: Clinical and economic burden of acute otitis media caused by Streptococcus pneumoniae in European children, after widespread use of PCVs–A systematic literature review of published evidence
Source: PLoS One. 2024 Apr 2;19(4):e0297098. doi: 10.1371/journal.pone.0297098 (PMC10986968; doi:10.1371/journal.pone.0297098)
Supplement: S4 Table — (DOCX) [file pone.0297098.s005.docx]

# Supporting information – Table S4

**S4 Table. Number of included records within each category, divided by country**

| **Country** | **Number of included records** | **Epidemiology** | ***S. pneumoniae*** | **Economic** | **Vaccine coverage** |
| --- | --- | --- | --- | --- | --- |
| Belgium | 3 | 2 | 3 |  | 3 |
| Bulgaria | 2 | 1 | 1 |  | 1 |
| Croatia | 1 | 1 |  | 1 |  |
| Cyprus | 1 |  |  |  | 1 |
| Denmark | 5 | 1 |  | 5 |  |
| Estonia | 1 | 1 |  | 1 |  |
| Europe | 3 | 3 |  | 1 | 2 |
| Finland | 8 | 8 | 2 | 3 | 1 |
| France | 15 | 10 | 13 |  | 5 |
| Germany | 11 | 7 | 2 | 8 | 2 |
| Greece | 1 | 1 |  | 1 |  |
| Hungary | 1 |  | 1 |  | 1 |
| Iceland | 6 | 6 | 1 | 1 | 1 |
| Italy | 13 | 9 | 2 | 6 | 6 |
| Lithuania | 1 | 1 |  | 1 |  |
| Netherlands | 8 | 7 |  | 5 | 2 |
| Norway | 4 | 4 | 1 | 2 | 2 |
| Poland | 4 | 4 | 1 | 1 |  |
| Portugal | 2 | 1 |  | 1 |  |
| Romania | 3 | 3 | 2 | 1 |  |
| Slovakia | 1 | 1 | 1 |  |  |
| Slovenia | 2 | 2 |  | 1 |  |
| Spain | 13 | 7 | 5 | 4 | 3 |
| Sweden | 16 | 8 | 1 | 9 | 5 |
| Switzerland | 3 | 2 | 1 | 2 | 2 |
| United Kingdom | 5 | 2 | 2 | 4 | 1 |
