# Supplementary material for: Clinical and economic burden of acute otitis media caused by Streptococcus pneumoniae in European children, after widespread use of PCVs–A systematic literature review of published evidence
Source: PLoS One. 2024 Apr 2;19(4):e0297098. doi: 10.1371/journal.pone.0297098 (PMC10986968; doi:10.1371/journal.pone.0297098)
Supplement: S5 Table — (DOCX) [file pone.0297098.s006.docx]

# Supporting information – Table S5

**S5 Table Records including antibiotic testing of S. pneumoniae in general and of specific serotypes**

| **Country** | **Number of studies with extracted antibiotic testing** | **Antibiotics tested** | **Including serotype-specific susceptibility** |
| --- | --- | --- | --- |
| Belgium [58, 73, 143] | 3 | Penicillin, Erythromycin, Multidrug resistance | 2 |
| Finland [47, 72] | 2 | Penicillin, Other antibiotics (incl. Macrolides) |  |
| France [11, 15, 59, 60, 66-68] | 8 | Penicillin, Ceftriaxone, Erythromycin, Clindamycin, Cotrimoxazole, Multidrug resistance |  |
| Germany [69] | 1 | Penicillin V, Betalactams |  |
| Iceland [63] | 1 | Penicillin, Ampicillin, Erythromycin, Sulphamethoxazole | 1 |
| Poland [53] | 1 | Penicillin, Ceftriaxone, TMP-SMX, Erythromycin, Clindamycin, Chloramphenicol, Tetracycline, Ceftriaxone, Multidrug resistance |  |
| Romania [55, 56] | 2 | Penicillin, Erythromycin, Tetracycline, Levofloxacin, Cotrimoxazole, Multidrug resistance | 2 |
| Sweden [64] | 1 | Penicillin, Cefotaxime, Erythromycin, Amoxicillin, Ampicillin, Cefpodoxime, Amoxicillin/clavulanic acid, Clindamycin, Tetracycline, TMP-SMX, Chloramphenicol, Ciprofloxacin, Multidrug resistance |  |
| Spain [57, 65, 70, 71] | 4 | Penicillin, Erythromycin | 2 |
| **Total** | **23** | **19** | **7** |
