# Supplementary material for: Clinical and economic burden of acute otitis media caused by Streptococcus pneumoniae in European children, after widespread use of PCVs–A systematic literature review of published evidence
Source: PLoS One. 2024 Apr 2;19(4):e0297098. doi: 10.1371/journal.pone.0297098 (PMC10986968; doi:10.1371/journal.pone.0297098)
Supplement: S6 Table — (DOCX) [file pone.0297098.s007.docx]

# Supporting information – Table S6

**S6 Table Summary data of the records that reported on the economic burden of AOM**

| **Country** | **Publication year** | **Population** | **Data source category** | **Cost categories reported** | | | **QoL measure** | **Reference** |
| --- | --- | --- | --- | --- | --- | --- | --- | --- |
|  |  |  |  | **Direct medical** | **Direct non-medical** | **Indirect** |  |  |
| **Economic modelling studies** | | | | | | | | |
| Belgium | 2019 | 0-48 months | Previously published literature | x | x | x |  | [19] |
| Croatia | 2015 | 1-59 months | Previously published literature | x | x |  | x | [142] |
| Denmark | 2013 | 0-12 months | Multiple sources | x |  |  |  | [120] |
| Finland | 2011 | 12-24 months | Previously published literature | x | x | x |  | [80] |
|  | 2020 | 0-24 months | Government data | x |  |  | x | [130] |
| France | 2019 | 0-48 months | Previously published literature | x | x | x |  | [19] |
| Germany | 2011 | 12-24 months | Previously published literature | x |  | x |  | [80] |
|  | 2012 | 0-204 months | Government data | x |  |  |  | [136] |
|  | 2015 | 0-72 months | Multiple sources | x |  | x | x | [108] |
|  | 2017 | 0-144 months | Multiple sources | x |  | x | x | [122] |
|  | 2019 | 0-48 months | Previously published literature | x | x | x |  | [19] |
| Greece | 2012 | Unspecified | Patient-level / cohort data | x |  |  |  | [136] |
| Italy | 2017 | Unspecified | Previously published literature | x |  |  |  | [36] |
|  | 2019 | 0-48 months | Previously published literature | x | x | x |  | [19] |
|  | 2020 | Unspecified | Multiple sources |  |  |  | x | [79] |
| Netherlands | 2019 | 0-48 months | Previously published literature | x | x | x |  | [19] |
| Norway | 2011 | 12-24 months | Previously published literature | x |  | x |  | [80] |
|  | 2011 | Unspecified | Previously published literature | x |  | x |  | [132] |
| Portugal | 2017 | Unspecified | Multiple sources | x |  | x |  | [117] |
|  | 2019 | 0-59 months | Previously published literature | x | x | x |  | [19] |
| Spain | 2011 | 0-120 months | Previously published literature | x | x | x |  | [80] |
|  | 2019 | 0-48 months | Previously published literature | x | x | x |  | [19] |
| Sweden | 2011 | 12-24 months | Previously published literature | x |  | x |  | [80] |
|  | 2012 | 0-108 months | Patient-level / cohort data | x |  | x |  | [105] |
|  | 2013 | 0-12 months | Previously published literature | x |  |  |  | [120] |
|  | 2019 | 0-59 months | Previously published literature | x | x | x |  | [19] |
| Switzerland | 2011 | 12-24 months | Previously published literature | x |  | x |  | [80] |
|  | 2012 | 0-60 months | Multiple sources | x |  |  |  | [104] |
| UK | 2012 | 0-192 months | Previously published literature | x |  |  | x | [99] |
|  | 2016 | Unspecified | Multiple sources | x |  | x | x | [110] |
|  | 2019 | 0-48 months | Previously published literature | x | x | x |  | [19] |
| Europe | 2019 | 0-60 months | Multiple sources | x | x | x | x | [106] |
| **Other types of studies** | | | | | | | | |
| Denmark | 2018 | Mean age: 16.3 months | Survey data |  |  |  | x | [119] |
|  | 2015 | 6-71 months | Survey data |  |  |  | x | [90] |
|  | 2014 | 6-72 months | Survey data |  |  |  | x | [91] |
|  | 2013 | Mean age: 16.4 months | Survey data |  |  |  | x | [88] |
| Estonia | 2016 | 3–71 months | Patient-level / cohort data | x |  | x |  | [30] |
| Finland | 2015 | 0-24 months | Patient-level / cohort data | x |  |  |  | [47] |
| Germany | 2020 | 0-192 months | Government data | x |  |  |  | [31] |
|  | 2019 | Unspecified | Insurance data | x |  |  |  | [133] |
|  | 2011 | 0-60 months | Survey data |  |  | x |  | [82] |
|  | 2015 | 0-60 months | Survey data |  |  |  | x | [93] |
| Iceland | 2020 | 0-36 months | Patient-level / cohort data | x |  |  | x | [34] |
| Italy | 2011 | 0-60 months | Survey data |  |  | x |  | [82] |
|  | 2015 | 0-60 months | Survey data |  |  |  | x | [93] |
|  | 2020 | 0-168 months | Patient-level / cohort data | x |  |  |  | [35] |
| Lithuania | 2016 | 0-71 months | Patient-level / cohort data |  |  | x |  | [30] |
| Netherlands | 2015 | 0-12 months | Patient-level / cohort data | x |  |  |  | [37] |
| Poland | 2016 | 0-71 months | Patient-level / cohort data | x |  | x |  | [30] |
| Romania | 2016 | 2–71 months | Patient-level / cohort data |  |  | x |  | [30] |
| Slovenia | 2016 | 1-70 months | Patient-level / cohort data | x |  | x |  | [30] |
| Spain | 2011 | 0-60 months | Survey data | x |  | x |  | [82] |
|  | 2015 | 0-60 months | Survey data |  |  |  | x | [93] |
|  | 2018 | 2-60 months | Patient-level / cohort data | x |  |  |  | [144] |
|  | 2021 | 0-24 months | Patient-level / cohort data | x |  |  |  | [39] |
|  | 2019 | Unspecified | Government data |  |  |  | x | [92] |
|  | 2011 | 0-60 months | Survey data | x |  | x |  | [82] |
|  | 2015 | 0-60 months | Survey data |  |  |  | x | [93] |
|  | 2011 | 0-60 months | Survey data | x | x | x |  | [83] |
| UK | 2011 | 0-60 months | Survey data | x |  | x |  | [82] |
|  | 2015 | 0-60 months | Survey data |  |  |  | x | [93] |
