# Supplementary material for: Clinical and economic burden of acute otitis media caused by Streptococcus pneumoniae in European children, after widespread use of PCVs–A systematic literature review of published evidence
Source: PLoS One. 2024 Apr 2;19(4):e0297098. doi: 10.1371/journal.pone.0297098 (PMC10986968; doi:10.1371/journal.pone.0297098)
Supplement: S7 Table — (DOCX) [file pone.0297098.s008.docx]

# Supporting information – Table S7

**S7 Table Definition groups for AOM for the included studies**

| **Number of papers** | **AOM definition category** |
| --- | --- |
| 1 | AOM treatment failure/recurrence |
| 1 | CDC-definition |
| 1 | Diagnosis in quality register |
| 1 | Diagnostic criteria (unspecified) |
| 11 | Doctor diagnosed |
| 19 | ICD-code(s) |
| 2 | ICPC-code |
| 3 | Multiple definitions |
| 32 | Not specified |
| 5 | Paradise criteria |
| 2 | Parent-reported |
| 4 | Pneumococcal isolate |
| 9 | Spontaneously draining/MEF presence/otorrhea/Suppurative |
| 3 | Spontaneous tympanic membrane perforation |
| 3 | Symptoms |
| 2 | Symptoms + otoscopic signs |
| 6 | Symptoms + Spontaneously draining / MEF presence / otorrhea / Suppurative |
| 2 | VT-scheduled |
