# Supplementary material for: Clinical and economic burden of acute otitis media caused by Streptococcus pneumoniae in European children, after widespread use of PCVs–A systematic literature review of published evidence
Source: PLoS One. 2024 Apr 2;19(4):e0297098. doi: 10.1371/journal.pone.0297098 (PMC10986968; doi:10.1371/journal.pone.0297098)
Supplement: Attachment — Submitted filename: COMMENTS on PONE-D-22-29637 - Leach.docx [file pone.0297098.s011.docx]

This review, titled “Clinical and economic burden of acute otitis media caused by Streptococcus pneumoniae in European children – a systematic literature review of published evidence” aimed to provide a current, comprehensive overview of AOM burden in Europe including economic burden, data on incidence, etiology, pneumococcal serotype and antibiotic resistance. The analyses provide trends in these outcomes over time (published since 2011) including PCV coverage and switching.

The authors are to be commended on a massive undertaking and well-presented summary of findings from 107 records detailed in 5 Tables, 7 Figures and 8 supporting documents.

I have made minor suggestions for the text, and somewhat greater changes suggested for some tables, to improve reader navigation of the data presented.

**Further recommendations**

The authors should consider making specific or explicit recommendations for future researchers reporting on this topic, such as the STROBE guidelines for (minimal data) reporting cross-sectional studies. (<https://www.strobe-statement.org/index.php?id=available-checklists>) see Table 8 in <https://doi.org/10.1016/j.ijporl.2019.109857> as a suggested format for recommended reporting on otitis media.

For microbiological studies of S. pneumoniae we recommend evaluation of methods against WHO recommendations (see comments for Table 4 and <https://doi.org/10.1016/j.vaccine.2013.08.062>) see also Table 4 in <https://doi.org/10.1016/j.ijporl.2019.109857>

**Abstract**

There is no mention of etiology, serotype shift or economic burden. To allow these findings to be included in the abstract word limit, lines 35 to 37 could be condensed. For instance, change to ‘.. wide variation in study methodology and reporting, which limited comparisons of all outcomes.’ Then delete ~~along with data gap~~ and delete sentence on L36-37 ‘Comparisons …methods.’

Line 39 after “depending on country” add “, PCV type and time since PCV introduction”)

Line 44 delete ~~due to variation in the methodologies~~ and finish with ‘improved by standardised methodology, reporting and ~~wider use of~~ surveillance systems’

**Introduction**

L54-55 change to “… caused by vaccine serotype S. pneumoniae.”

L60 change have to has

L62 change new to non-vaccine (the serotypes are not ‘new’, non-vaccine serotypes emerge as the vaccine serotypes are eliminated)

L67-68 suggest change wording after ; to ;’ in addition to the serotypes present in PCV13, PCV15 contains 22F and 33F, and PCV20 contains serotypes 8, 10A, 11A, 12F, and 15B’

L84 remove comma after collecting

L85 add ‘of age’ after < 5 years

**Methods**

L92 I could not access the PROSPERO hyperlink in Chrome or Edge

L112-115 Why were abstracts from ISPPD International Symposium on Pneumococci and Pneumococcal Diseases not included as highly relevant.

L125 add reference to your PRISMA flowdiagram Figure 1 – although I have noted that this is illegible in the PDF.

L145 change ‘this type of studies’ to ‘this type of study’

L164 change ‘data was’ to ‘data were’ (data are plural)

**Results**

L185 Figure 1 PRISMA flowchart is illegible

L191 change ‘…category, 19 of them…’ to ‘…category, only 19 of them…’ (as this sentence commenced with ‘Even though..)

L196 could delete ‘and onwards’

L201 Start with ‘Of the 61 studies ..’ (i.e. delete ‘Out’)

L217 change ‘incidence different’ to ‘incidence were different’

L221 Delete ‘However’ and start sentence with Data

L226 to 230 – include in this section the different vaccine eras and time since PCV introduced or switched.

L237 provide range in proportion positive for both MEF and NP. Change to ‘… ranged from 5.8% to 70% in MEF samples .. in Germany and Romania, respectively and from 10.5% to 83% in nasopharyngeal (NP) samples … in Sweden and Portugal, respectively’

L246 Please elaborate on differences in sampling methods and influence on country-specific data for MEF and NP.

L248 – section on antibiotic resistance – should be reported for MEF and for NP samples as resistance can be quite different according to site (as also noted in section on serotype distribution)

L261 Delete ‘Again’ and structure sentence to read the same as first sentence (L258). Ie “There was a ***lower*** level of serotype-specific non-susceptibility in the **post**-PCV time periods”

L264 Changing Figure S1 as suggested below will help understand mid-PCV period and years of PCV switch

L283 It is important to recognise and distinguish between date of publication and date of data collection (as has been done elsewhere).

L284 change ‘contained data from 2011’ to ‘contained data **published** from 2011’

Line 426 **Funding**. It is noteworthy that this review was commissioned and conducted by Merck Sharp & Dome who are developing expanded valency pneumococcal conjugate vaccines. Please include ***role*** of funders in design, analysis, decision to publish. Author contributions on page 1. It appears that MSD conceptualized and developed design, and had input into extraction, synthesis and writing of the manuscript. Please clarify.

**TABLES and FIGURES**

**Figure 4**

is illegible

**Figure 5**

add N to each bar, proportions alone are not very informative. X-axis is illegible.

**Figures 6 and 7**

These two Figures could be combined which would show the country differences in proportion of total cost that is direct cost.

**Table 1**

For conference abstracts add final date

**Table 2**

Column ‘Time interval’ – add PCV type, year of introduction or switch (e.g. 2011. PCV10. 2001). Perhaps change column title from ‘Time interval’ to ‘Period of data collection’

**Table 3**

Consider rounding decimal places. Add which PCV in Columns pre-PCV (or no PCV) Post-PCV (Iceland used PCV10). Reference 36 title states “transition era …PCV7 to PCV13.., 2010 to 2013”. Are the data presented in the table pre- and post-, excluding transition era?

**Table 4**

Column Time interval – add PCV (PCV10 or PCV13), and year of PCV introduction (e.g. 2011. PCV10. 2001). Perhaps change column title from ‘Time interval’ to ‘Period of data collection’

Sampling method can be the major explanation for differences in detection of pneumococci – consider adding a column for ‘Sampling Method’. This could be culture vs PCR, or compliance with WHO standard method (O'Brien,K.L and Nohynek,H. 2003. Report from a WHO working group: standard method for detecting upper respiratory carriage of Streptococcus pneumoniae OR Satzke, C. et al. 2013 Standard method for detecting upper respiratory carriage of Streptococcus pneumoniae: Updated recommendations from the World Health Organization Pneumococcal Carriage Working Group) 10.1016/j.vaccine.2013.08.062.)

95% Confidence Intervals for the point estimates would be helpful, given huge differences in sample size. Column Estimate (%, 95%CI)

**Table 5**

Column Time interval – add PCV (PCV10 or PCV13), and year of PCV introduction (e.g. 2011. PCV10. 2001). Perhaps change column title from ‘Time interval’ to ‘Period of data collection’

Column Population – add N for each age range and year (e.g. 0-23 months. N)

The row totals for PCV7 serotypes, PCV13 serotypes are incomplete, and would be helpful to include.

Add a final column for row total of non-PCV serotypes (i.e. 100 — (PCV13+PCV15 +PCV20))

**SUPPORTING DOCUMENTS**

**Table S3**

As year of publication is given in Column 1, change column ‘Year of publication’ to ‘Year of data collection’

**Table S5**

Column *S. pneumoniae* could you split this into detection method (culture, PCR) and serotype (Y/N)

**Table S7**

the grey highlights are not visible

**Figure S1**

Instead of yes, I suggest inserting PCV type i.e. PCV7, PCV10 or PCV13. [this will help track country differences in PCV and switch between PCVs – as noted L211 to 223, page 11 and also L265-266, page 15]

**Further recommendations**

The authors should consider making specific or explicit recommendations for future researchers reporting on this topic, such as the STROBE guidelines for (minimal data) reporting cross-sectional studies. (<https://www.strobe-statement.org/index.php?id=available-checklists>) see Table 8 in <https://doi.org/10.1016/j.ijporl.2019.109857> as a suggested format for recommended reporting on otitis media.

For microbiological studies of S. pneumoniae we recommend evaluation of methods against WHO recommendations (see comments for Table 4 and <https://doi.org/10.1016/j.vaccine.2013.08.062>) see also Table 4 in <https://doi.org/10.1016/j.ijporl.2019.109857>
