# Supplementary material for: Clinical and economic burden of acute otitis media caused by Streptococcus pneumoniae in European children, after widespread use of PCVs–A systematic literature review of published evidence
Source: PLoS One. 2024 Apr 2;19(4):e0297098. doi: 10.1371/journal.pone.0297098 (PMC10986968; doi:10.1371/journal.pone.0297098)
Supplement: Attachment — Submitted filename: AOM SLR resubmission_Response to Reviewers_v1_20230119.docx [file pone.0297098.s012.docx]

Dear reviewers,

Thank you for providing us with a thorough review of our manuscript. We have gone through the comments and have done our best to address all the thoughtful input you have provided.

In the table below, you will find the complete list of all your comments and how each of them was addressed. Please be aware that the line numbers refer to the previous version and it is likely that they have changed in the revised manuscript.

We would also like to note that since many of the comments discussed relating the data to when PCVs were introduced in the included countries, we made significant adjustments to the tables and figures to accommodate that information. We have also added a bit more data related to non-vaccine serotypes to further expand upon the concept of residual burden. We hope that these aspects clarify and strengthen our conclusions from the collected data, to provide your readership with a better picture of the burden of AOM after widespread use of PCVs in Europe.

| **Section** | **Reviewer** | **Reviewer’s comment** | **Author’s response** |
| --- | --- | --- | --- |
| N/A | 1 | AOM burden is quantified by the number of AOM cases/recurrent AOM cases, and not by the number of AOM hospitalizations or antibiotic resistance patterns of bacteria isolated from AOM cases, which becomes more and more rare since myringotomy is not routinely performed anymore.  I think that the authors worked pretty hard on this manuscript, and I will strongly encourage them to revise it. | Due to the data heterogeneity in AOM studies, we could not identify a common pattern in AOM burden for all countries over time. However, we see AOM hospitalizations and antibiotic resistance patterns in S. pneumoniae as relevant aspects that contribute especially to the economic burden of AOM. This was added to the Discussion. |
| Abstract | 1 | The abstract should be clearer: which PCVs are included in this review? the results given- to which vaccine? from 2011 there are many good quality papers that cover PCV7, PHiD-CV (PCV10) and PCV13. This distinction should be clearly stated and referred to. What were your criteria and outcomes in your research? The conclusions part is very general and not based on your results—when you say that AOM burden in Europe is still very high—you did not give any numbers in the previous Results paragraph. | We have added further information about these aspects in the abstract. |
|  | 2 | There is no mention of etiology, serotype shift or economic burden. To allow these findings to be included in the abstract word limit, lines 35 to 37 could be condensed. For instance, change to ‘.. wide variation in study methodology and reporting, which limited comparisons of all outcomes.’ Then delete ~~along with data gap~~ and delete sentence on L36-37 ‘Comparisons …methods.’ | We have now mentioned these aspects and our findings in the abstract, and condensed the appropriate lines. |
|  | 2 | Line 39 after “depending on country” add “, PCV type and time since PCV introduction”) | Implemented as suggested by reviewer |
|  | 2 | Line 44 delete ~~due to variation in the methodologies~~ and finish with ‘improved by standardised methodology, reporting and ~~wider use of~~ surveillance systems’ | Implemented as suggested by reviewer |
| Introduction | 1 | Give PHiD-CV and PCV13 timelines. PCV15/PCV20—mention them in the Discussion section. Redefine your research question, it still remains unclear. |  |
|  | 2 | L54-55 change to “… caused by vaccine serotype S. pneumoniae.” | Implemented as suggested by reviewer |
|  | 2 | L60 change have to has | Implemented as suggested by reviewer |
|  | 2 | L62 change new to non-vaccine (the serotypes are not ‘new’, non-vaccine serotypes emerge as the vaccine serotypes are eliminated) | Implemented as suggested by reviewer |
|  | 2 | L67-68 suggest change wording after ; to ;’ in addition to the serotypes present in PCV13, PCV15 contains 22F and 33F, and PCV20 contains serotypes 8, 10A, 11A, 12F, and 15B’ | Implemented as suggested by reviewer |
|  | 2 | L84 remove comma after collecting | Implemented as suggested by reviewer |
|  | 2 | L85 add ‘of age’ after < 5 years | Implemented as suggested by reviewer |
| Methods | 1 | Methods can be shortened. Leave only what's important. How did you actually assess the change in AOM burden after PCVs were available? | Change in burden was assessed by comparing estimates before and after introduction of PCVs. This was added to the Methods section. |
|  | 2 | L92 I could not access the PROSPERO hyperlink in Chrome or Edge | The link was replaced and should work now |
|  | 2 | L112-115 Why were abstracts from ISPPD International Symposium on Pneumococci and Pneumococcal Diseases not included as highly relevant. | The abstract search was a complement to the database searches (PubMed and Embase) and was therefore limited to 3 conferences. Due do the geographic scope of this study, a priority was given to European conferences. Additionally, at the time of SLR, the last ISPPD was in 2020. So, it was assumed that in 1,5 years most of the abstracts were published as manuscripts. |
|  | 2 | L125 add reference to your PRISMA flowdiagram Figure 1 – although I have noted that this is illegible in the PDF. | Reference to Fig1 was included |
|  | 2 | L145 change ‘this type of studies’ to ‘this type of study’ | Implemented as suggested by reviewer |
|  | 2 | L164 change ‘data was’ to ‘data were’ (data are plural) | Implemented as suggested by reviewer |
| Results | 1 | Results. It is essential to state for each paper that you show in Table 2 what was the immunization status of the children-vaccinated (with what?), unvaccinated? so we can understand the figures. Do you have any pre-vaccine data for comparison purposes?. It appears that you missed many papers from France. Here are a few of them that may be useful:  - Bacterial causes of otitis media with spontaneous perforation of the tympanic membrane in the era of the 13-valent pneumococcal conjugate vaccine.  Levy C, Varon E, Ouldali N, Wollner A, Thollot F, Corrard F, Werner A, Béchet S, Bonacorsi S, Cohen R.  PLoS One. 2019 Feb 1;14(2):e0211712. doi: 10.1371/journal.pone.0211712.  - The multifaceted impact of pneumococcal conjugate vaccine implementation in children in France between 2001 to 2014.  Cohen R, Biscardi S, Levy C.  Hum Vaccin Immunother. 2016;12(2):277-84. doi: 10.1080/21645515.2015.1116654.  - Community antibiotic prescribing for children in France from 2015 to 2017: a cross-sectional national study.  Trinh NTH, Cohen R, Lemaitre M, Chahwakilian P, Coulthard G, Bruckner TA, Milic D, Levy C, Chalumeau M, Cohen JF.  J Antimicrob Chemother. 2020 Aug 1;75(8):2344-2352. doi: 10.1093/jac/dkaa162.  - Antibiotic Resistance of Potential Otopathogens Isolated From Nasopharyngeal Flora of Children With Acute Otitis Media Before, During and After Pneumococcal Conjugate Vaccines Implementation.  Rybak A, Levy C, Bonacorsi S, Béchet S, Vié le Sage F, Elbez A, Varon E, Cohen R.  Pediatr Infect Dis J. 2018 Mar;37(3):e72-e78. doi: 10.1097/INF.0000000000001862. | Thank you for mentioning these articles. Rybak et al, 2018, Cohen et al. 2016 and Levy et al. 2019 were actually included in Table 4 (references 83, 96 and 97, respectively).  Based on your suggestion, we reviewed Trinh et al. 2020. We believe that this article did not meet the inclusion criteria as it was not possible to differentiate the antibiotic prescriptions that were specific for AOM from the other types of otitis. |
|  | 2 | L185 Figure 1 PRISMA flowchart is illegible | The figure has been adjusted and re-submitted |
|  | 2 | L191 change ‘…category, 19 of them…’ to ‘…category, only 19 of them…’ (as this sentence commenced with ‘Even though..) | Implemented as suggested by reviewer |
|  | 2 | L196 could delete ‘and onwards’ | Implemented as suggested by reviewer |
|  | 2 | L201 Start with ‘Of the 61 studies ..’ (i.e. delete ‘Out’) | Implemented as suggested by reviewer |
|  | 2 | L217 change ‘incidence different’ to ‘incidence were different’ | Implemented as suggested by reviewer |
|  | 2 | L221 Delete ‘However’ and start sentence with Data | Implemented as suggested by reviewer |
|  | 2 | L226 to 230 – include in this section the different vaccine eras and time since PCV introduced or switched. | Described the results within the context of PCV introduction for each country with relevant data |
|  | 2 | L237 provide range in proportion positive for both MEF and NP. Change to ‘… ranged from 5.8% to 70% in MEF samples .. in Germany and Romania, respectively and from 10.5% to 83% in nasopharyngeal (NP) samples … in Sweden and Portugal, respectively’ | Implemented as suggested by reviewer |
|  | 2 | L246 Please elaborate on differences in sampling methods and influence on country-specific data for MEF and NP. | The differences have been elaborated upon |
|  | 2 | L248 – section on antibiotic resistance – should be reported for MEF and for NP samples as resistance can be quite different according to site (as also noted in section on serotype distribution) | Nearly all studies assessed antibiotic resistance in MEF samples, except for one. This information was added to the manuscript. |
|  | 2 | L261 Delete ‘Again’ and structure sentence to read the same as first sentence (L258). Ie “There was a ***lower*** level of serotype-specific non-susceptibility in the **post**-PCV time periods” | Implemented as suggested by reviewer |
|  | 2 | L264 Changing Figure S1 as suggested below will help understand mid-PCV period and years of PCV switch | This has been fixed. Please see comment below |
|  | 2 | L283 It is important to recognise and distinguish between date of publication and date of data collection (as has been done elsewhere). | Text was modified to specify that it referred to the data collection period |
|  | 2 | L284 change ‘contained data from 2011’ to ‘contained data **published** from 2011’ | Text was modified to specify that it referred to the data collection period |
| Discussion / Conclusion | 1 | By generally describing that "PCVs" reduced AOM, it is hard to figure out which one of them was a key player.  I highly recommend the authors to look at other non-European studies where there is a clear differentiation between each PCV. This truly determines the epidemiology and bacteriology of AOM nowadays. Resistance of pneumococci- again, it is mandatory to state whether there were bacteria grown in ear cultures or from nasopharyngeal swabs. as the concordance rate between the nasopharynx and the middle ear is only moderate...  Discussion is nicely written, but the Conclusion parts is too general | We have now added additional information to the manuscript regarding the specific PCVs present, as well as further connection between the available data and PCVs present in the countries at the time.  For this review, non-European studies were out of scope for the study. This would however be a very interesting area for further research. Thank you for the suggestion. The sampling sources have been clarified for the outcomes where it is relevant. |
| Funding | 2 | Line 426 **Funding**. It is noteworthy that this review was commissioned and conducted by Merck Sharp & Dome who are developing expanded valency pneumococcal conjugate vaccines. Please include ***role*** of funders in design, analysis, decision to publish. Author contributions on page 1. It appears that MSD conceptualized and developed design, and had input into extraction, synthesis and writing of the manuscript. Please clarify. | The funding information was described in the Financial Disclosure Statement according to PLOS ONE requirements. |
| TABLES and FIGURES | | | |
| Figure 4 | N/A | 7. We note that Figure 4 in your submission contain map images which may be copyrighted. All PLOS content is published under the Creative Commons Attribution License (CC BY 4.0), which means that the manuscript, images, and Supporting Information files will be freely available online, and any third party is permitted to access, download, copy, distribute, and use these materials in any way, even commercially, with proper attribution. For these reasons, we cannot publish previously copyrighted maps or satellite images created using proprietary data, such as Google software (Google Maps, Street View, and Earth). For more information, see our copyright guidelines: <http://journals.plos.org/plosone/s/licenses-and-copyright>.    We require you to either (1) present written permission from the copyright holder to publish these figures specifically under the CC BY 4.0 license, or (2) remove the figures from your submission:  (1) You may seek permission from the original copyright holder of Figure 4 to publish the content specifically under the CC BY 4.0 license.    We recommend that you contact the original copyright holder with the Content Permission Form (<http://journals.plos.org/plosone/s/file?id=7c09/content-permission-form.pdf>) and the following text:  “I request permission for the open-access journal PLOS ONE to publish XXX under the Creative Commons Attribution License (CCAL) CC BY 4.0 (<http://creativecommons.org/licenses/by/4.0/>). Please be aware that this license allows unrestricted use and distribution, even commercially, by third parties. Please reply and provide explicit written permission to publish XXX under a CC BY license and complete the attached form.”    Please upload the completed Content Permission Form or other proof of granted permissions as an ""Other"" file with your submission.    In the figure caption of the copyrighted figure, please include the following text: “Reprinted from [ref] under a CC BY license, with permission from [name of publisher], original copyright [original copyright year].”  (2) If you are unable to obtain permission from the original copyright holder to publish these figures under the CC BY 4.0 license or if the copyright holder’s requirements are incompatible with the CC BY 4.0 license, please either i) remove the figure or ii) supply a replacement figure that complies with the CC BY 4.0 license. Please check copyright information on all replacement figures and update the figure caption with source information. If applicable, please specify in the figure caption text when a figure is similar but not identical to the original image and is therefore for illustrative purposes only.  The following resources for replacing copyrighted map figures may be helpful:    USGS National Map Viewer (public domain): <http://viewer.nationalmap.gov/viewer/>  The Gateway to Astronaut Photography of Earth (public domain): <http://eol.jsc.nasa.gov/sseop/clickmap/>  Maps at the CIA (public domain): <https://www.cia.gov/library/publications/the-world-factbook/index.html> and <https://www.cia.gov/library/publications/cia-maps-publications/index.html>  NASA Earth Observatory (public domain): <http://earthobservatory.nasa.gov/>  Landsat: <http://landsat.visibleearth.nasa.gov/>  USGS EROS (Earth Resources Observatory and Science (EROS) Center) (public domain): [http://eros.usgs.gov/#](http://eros.usgs.gov/)  Natural Earth (public domain): <http://www.naturalearthdata.com/> |  |
|  |  |  | Thank you very much for providing some suggested alternative sources for map figures. We understand the journal’s reservation in publishing copyrighted materials.  We have therefore opted to use a bubble chart to display the information about changes to antibiotic resistance over time. This allows us to incorporate a further dimension that is sample size to the figure. |
|  | 2 | is illegible | Thank you for noticing that. The figure has been adjusted and re-submitted |
| Figure 5 | 2 | add N to each bar, proportions alone are not very informative. X-axis is illegible. | Figure has been adjusted to include the n for each bar and increase legibility for the x axis |
| Figures 6 and 7 | 2 | These two Figures could be combined which would show the country differences in proportion of total cost that is direct cost. | Figures 6 and 7 have been combined. The accompanying text has been adjusted to match |
| Table 1 | 2 | For conference abstracts add final date | Dates were added to Table 1 |
| Table 2 | 2 | Column ‘Time interval’ – add PCV type, year of introduction or switch (e.g. 2011. PCV10. 2001). Perhaps change column title from ‘Time interval’ to ‘Period of data collection’ | PCV column was added, estimates related to hospitalization incidence in table 2 were removed, as they were not relevant for the context of the table. Title of column 3 was changed per your suggestion |
| Table 3 | 2 | Consider rounding decimal places. Add which PCV in Columns pre-PCV (or no PCV) Post-PCV (Iceland used PCV10). Reference 36 title states “transition era …PCV7 to PCV13.., 2010 to 2013”. Are the data presented in the table pre- and post-, excluding transition era? | Decimal places were rounded. PCVs were specified to clarify the transition |
| Table 4 | 2 | Column Time interval – add PCV (PCV10 or PCV13), and year of PCV introduction (e.g. 2011. PCV10. 2001). Perhaps change column title from ‘Time interval’ to ‘Period of data collection’ | PCV per time period was added to the table. Time interval heading changed. |
|  | 2 | Sampling method can be the major explanation for differences in detection of pneumococci – consider adding a column for ‘Sampling Method’. This could be culture vs PCR, or compliance with WHO standard method (O'Brien,K.L and Nohynek,H. 2003. Report from a WHO working group: standard method for detecting upper respiratory carriage of Streptococcus pneumoniae OR Satzke, C. et al. 2013 Standard method for detecting upper respiratory carriage of Streptococcus pneumoniae: Updated recommendations from the World Health Organization Pneumococcal Carriage Working Group) 10.1016/j.vaccine.2013.08.062.) | We appreciate your valuable input that the sampling method can affect the detection of *S.pneumoniae*. However, the WHO recommendation is limited to NP samples, i.e., do not comprise MEF, which is also relevant for AOM. Furthermore, analysing the differences between sampling methods was not in the scope of our review. Due to these reasons, we did not include this information in Table 4. |
|  | 2 | 95% Confidence Intervals for the point estimates would be helpful, given huge differences in sample size. Column Estimate (%, 95%CI) | According to the data we extracted from the included records, only one study reported a confidence interval for the aetiology estimates. We included the single interval in table 4. |
| Table 5 | 2 | Column Time interval – add PCV (PCV10 or PCV13), and year of PCV introduction (e.g. 2011. PCV10. 2001). Perhaps change column title from ‘Time interval’ to ‘Period of data collection’ | PCVs added, Column heading changed |
|  | 2 | Column Population – add N for each age range and year (e.g. 0-23 months. N) | N has been added in the age range column for each age range and year, by order of publication |
|  | 2 | The row totals for PCV7 serotypes, PCV13 serotypes are incomplete, and would be helpful to include. | The columns for PCV serotypes are not totals but instead provide estimates from studies that did not report the specific serotypes but instead grouped the vaccine serotypes into one total. |
|  | 2 | Add a final column for row total of non-PCV serotypes (i.e. 100 — (PCV13+PCV15 +PCV20)) | We have added a new table with the information for non-PCV serotypes and expanded upon the results section to tie this information to residual burden |
| SUPPORTING DOCUMENTS | | | |
| Table S3 | 2 | As year of publication is given in Column 1, change column ‘Year of publication’ to ‘Year of data collection’ | The column has been added |
| Table S5 | 2 | Column *S. pneumoniae* could you split this into detection method (culture, PCR) and serotype (Y/N) | Since renumbering the tables, this comment is now relevant for table S4. The table focuses on a general overview of the data categories available for each country within the subheadings of the manuscript. Given the general nature of this table’s content, dividing the S. pneumoniae column up by detection method and serotype would both likely create confusion, as well as providing redundant information that other tables describe or be out of scope for the study itself. |
| Table S7 | 2 | the grey highlights are not visible | Thank for noticing that. The grey highlight had actually been removed. Instead, the records were divided into “Economic modelling studies” and “Other types of studies”. The table heading has been updated. |
| Figure S1 | 2 | Instead of yes, I suggest inserting PCV type i.e. PCV7, PCV10 or PCV13. [this will help track country differences in PCV and switch between PCVs – as noted L211 to 223, page 11 and also L265-266, page 15] | We have redone figure S1 to include the specific PCVs for each country by year |
|  | N/A | Please upload a new copy of Figure S1 as the detail is not clear. Please follow the link for more information:  <https://blogs.plos.org/plos/2019/06/looking-good-tips-for-creating-your-plos-figures-graphics/>  <https://blogs.plos.org/plos/2019/06/looking-good-tips-for-creating-your-plos-figures-graphics/> | The figure was adjusted and re-submitted |
| OTHER | | | |
| Further recommendations | 2 | The authors should consider making specific or explicit recommendations for future researchers reporting on this topic, such as the STROBE guidelines for (minimal data) reporting cross-sectional studies. (<https://www.strobe-statement.org/index.php?id=available-checklists>) see Table 8 in <https://doi.org/10.1016/j.ijporl.2019.109857> as a suggested format for recommended reporting on otitis media.  For microbiological studies of S. pneumoniae we recommend evaluation of methods against WHO recommendations (see comments for Table 4 and <https://doi.org/10.1016/j.vaccine.2013.08.062>) see also Table 4 in <https://doi.org/10.1016/j.ijporl.2019.109857> | Thank you for your suggestion, it is indeed a valid point and we have incorporated it in our conclusions. |
| JOURNAL REQUIREMENTS | | | |
| Style requirements |  | 1. Please ensure that your manuscript meets PLOS ONE's style requirements, including those for file naming. The PLOS ONE style templates can be found at  <https://journals.plos.org/plosone/s/file?id=wjVg/PLOSOne_formatting_sample_main_body.pdf> and  <https://journals.plos.org/plosone/s/file?id=ba62/PLOSOne_formatting_sample_title_authors_affiliations.pdf> | We reviewed our manuscript so it matches with the style requirements |
| Funding information |  | 2. We note that the grant information you provided in the ‘Funding Information’ and ‘Financial Disclosure’ sections do not match.  When you resubmit, please ensure that you provide the correct grant numbers for the awards you received for your study in the ‘Funding Information’ section. | Thank you for your comment. As per PLOS ONE requirements, the funding information has been removed from the manuscript and it is now available only in the submission system. For information, no grants or awards were received to conduct this study. |
| Financial disclosure |  | 3. Please expand the acronym “MSD” (as indicated in your financial disclosure) so that it states the name of your funders in full.  This information should be included in your cover letter; we will change the online submission form on your behalf. | The acronym has been explained and it is now mentioned in the cover letter as well. |
|  |  | 4. Thank you for stating the following financial disclosure:  "This study was funded by MSD. ET reviewed the study design. ET and GB reviewed the data collection and analysis, as well as the decision to publish. GB reviewed the preparation of the manuscript."  At this time, please address the following queries:  a) Please clarify the sources of funding (financial or material support) for your study. List the grants or organizations that supported your study, including funding received from your institution.  b) State what role the funders took in the study. If the funders had no role in your study, please state: “The funders had no role in study design, data collection and analysis, decision to publish, or preparation of the manuscript.”  c) If any authors received a salary from any of your funders, please state which authors and which funders.  d) If you did not receive any funding for this study, please state: “The authors received no specific funding for this work.”  Please include your amended statements within your cover letter; we will change the online submission form on your behalf. | The financial disclosure has been adjusted as requested. The updated text has been included in the cover letter and in the submission system. |
| Competing interests | N/A | 5. Thank you for stating the following in the Competing Interests section:  "I have read the journal's policy and the authors of this manuscript have the following competing interests: ET and GB are employees of MSD. HRC, HS and NN are employees of Quantify Research, which received consulting fees from MSD to support the preparation and development of the manuscript. The authors report no other conflicts of interest in this work."    We note that one or more of the authors are employed by a commercial company: MSD, Quantify Research  (1) Please provide an amended Funding Statement declaring this commercial affiliation, as well as a statement regarding the Role of Funders in your study. If the funding organization did not play a role in the study design, data collection and analysis, decision to publish, or preparation of the manuscript and only provided financial support in the form of authors' salaries and/or research materials, please review your statements relating to the author contributions, and ensure you have specifically and accurately indicated the role(s) that these authors had in your study. You can update author roles in the Author Contributions section of the online submission form.  Please also include the following statement within your amended Funding Statement.  “The funder provided support in the form of salaries for authors [insert relevant initials], but did not have any additional role in the study design, data collection and analysis, decision to publish, or preparation of the manuscript. The specific roles of these authors are articulated in the ‘author contributions’ section.”  If your commercial affiliation did play a role in your study, please state and explain this role within your updated Funding Statement.  (2) Please also provide an updated Competing Interests Statement declaring this commercial affiliation along with any other relevant declarations relating to employment, consultancy, patents, products in development, or marketed products, etc.  Within your Competing Interests Statement, please confirm that this commercial affiliation does not alter your adherence to all PLOS ONE policies on sharing data and materials by including the following statement: ""This does not alter our adherence to PLOS ONE policies on sharing data and materials.” (as detailed online in our guide for authors <http://journals.plos.org/plosone/s/competing-interests>).  If this adherence statement is not accurate and there are restrictions on sharing of data and/or materials, please state these. Please note that we cannot proceed with consideration of your article until this information has been declared.  Please include both an updated Funding Statement and Competing Interests Statement in your cover letter. We will change the online submission form on your behalf. | Thank you for your comments. The Financial Disclosure and the Competing Interests statements have been updated accordingly and are provided in the cover letter. |
| Data availability |  | 6. We note that you have indicated that data from this study are available upon request. PLOS only allows data to be available upon request if there are legal or ethical restrictions on sharing data publicly. For more information on unacceptable data access restrictions, please see <http://journals.plos.org/plosone/s/data-availability#loc-unacceptable-data-access-restrictions>.  In your revised cover letter, please address the following prompts:  a) If there are ethical or legal restrictions on sharing a de-identified data set, please explain them in detail (e.g., data contain potentially sensitive information, data are owned by a third-party organization, etc.) and who has imposed them (e.g., an ethics committee). Please also provide contact information for a data access committee, ethics committee, or other institutional body to which data requests may be sent.  b) If there are no restrictions, please upload the minimal anonymized data set necessary to replicate your study findings as either Supporting Information files or to a stable, public repository and provide us with the relevant URLs, DOIs, or accession numbers. For a list of acceptable repositories, please see <http://journals.plos.org/plosone/s/data-availability#loc-recommended-repositories>.  We will update your Data Availability statement on your behalf to reflect the information you provide. |  |
| PRISMA Checklist |  | 9. We note that this manuscript is a systematic review or meta-analysis; our author guidelines therefore require that you use PRISMA guidance to help improve reporting quality of this type of study. Please upload copies of the completed PRISMA checklist as Supporting Information with a file name “PRISMA checklist”. | The PRISMA checklist had been submitted in the Supporting Information file, it has now been re-submitted as a stand-alone file |
