# Supplementary material for: Clinical and economic burden of acute otitis media caused by Streptococcus pneumoniae in European children, after widespread use of PCVs–A systematic literature review of published evidence
Source: PLoS One. 2024 Apr 2;19(4):e0297098. doi: 10.1371/journal.pone.0297098 (PMC10986968; doi:10.1371/journal.pone.0297098)
Supplement: Attachment — Submitted filename: AOM SLR_2nd Response to Reviewers_v1_202304228.docx [file pone.0297098.s013.docx]

Dear reviewers,

Thank you for providing us with a second review of our manuscript. We have gone through the comments and have done our best to address the thoughtful input you have provided.

In the table below, you will find the complete list of the second round of comments and how each of them was addressed. Please be aware that the line numbers refer to the previous version and it is likely that they have changed in the revised manuscript. However, any changes made to the text and tables can also be found in the ‘track changes’ version of the manuscript, where we have also highlighted those changes in yellow and added a comment explaining the change, in addition to tracking them.

We hope that the revisions clarify and strengthen our conclusions from the collected data, to provide your readership with a better picture of the burden of AOM after widespread use of PCVs in Europe.

| **Section** | **Reviewer** | **Reviewer’s comment** | **Response** |
| --- | --- | --- | --- |
| Journal requirements | journal | Please review your reference list to ensure that it is complete and correct. If you have cited papers that have been retracted, please include the rationale for doing so in the manuscript text, or remove these references and replace them with relevant current references. Any changes to the reference list should be mentioned in the rebuttal letter that accompanies your revised manuscript. If you need to cite a retracted article, indicate the article’s retracted status in the References list and also include a citation and full reference for the retraction notice. | We have gone through the references to make sure they align with the bibliography at the end, removing any duplicate references. No papers have been retracted. |
| General comments | 1 | I can't see your corrections. Please outline them and refer to the comments you have received by the reviewers. Thank you | For this round of comments, we have highlighted any changes in yellow and included a comment explaining the reason for the change, in addition to applying the track-changes function. |
|  | 3 | This study is very important that it summarized the most recent AOM burden in EU countries comprehensively. I only have a few minor comments for the author to consider: | Thank you so much! We hope our revisions appropriately address the comments you’ve added |
| Content comments | 3 | Line 78-83: what is the age range for this incidence rate? And spell out xx cases per 1000 person years. And which year are the incidence rates in this paragraph for? Is it pre or post PCV introduction? | We added an explanation of age in the estimate, the year of the estimate and converted the estimate to 1000 person-years. The underlying study did not specify about vaccination status for this global estimate, so we assume that the estimate encompasses both unvaccinated and vaccinated people around the world. |
|  | 3 | Line 81-82: Could you clarify this? Is the incidence rate higher in the <1 or 1-4 years old? | We rephrased the sentence to explain that it was patients between 0-4 years of age. |
|  | 3 | Table 1 and other appropriate sections related with the name "economic burden": QoL is humanistic burden, suggest expanding the name to accurately capture the outcomes that this study assessed | We have edited the table and relevant section headings to avoid the confusion around economic and humanistic burden. |
|  | 3 | Line 248: Is this inpatient stay? Compared to inpatient stay, AOM is more likely to be associated with outpatient visits and the HCRU associated with outpatient/physician office visits is a greater burden to the health care system. Suggest look into the literature on physician office visits, or provide explanations or plan to look into this in the future. | We clarified in the text that hospitalization is referring to inpatient stay. We also went back to the included records that provided estimates for hospitalization rates to see if they also included rates for outpatient visits. Unfortunately, those records did not contain relevant estimates. However, the economic burden related to outpatient visits is discussed in the economic burden section, in the form of direct costs associated with AOM. So we feel that this aspect of the burden is represented in the findings, even if we didn’t identify office-visit rates in the collected literature. |
|  | 3 | Line 263-264: suggest putting some context around those percentages, are those from pre or post PCV adoption period for those countries? | Further context related to the PCV adoption period has been added to the estimates provided in the text. |
|  | 3 | Line 270: any possible explanations for the difference? Different regions, methods for taking samples, testing method difference? | We provided an explanation for the differences in estimates, which can in part be explained by differences in the AOM populations studied. |
|  | 3 | Line 396: would be interesting to look at other contributing factors such as physician office visits, and antibiotic prescriptions in addition to the antibiotic resistance | We agree that these factors would be interesting to look into, as they would also provide evidence of how the burden of AOM may have shifted across Europe. Unfortunately, they were not within the scope of the review, and would require a significant change to the scope of the study. We therefore decided to instead include a sentence in the limitations regarding the factors you suggested. |
